# Essential complicity of perforin-granzyme and FAS-L mechanisms to achieve tumor rejection following treatment with anti-CD137 mAb

**DOI:** 10.1186/2051-1426-1-3

**Published:** 2013-05-29

**Authors:** Aizea Morales-Kastresana, Elena Catalán, Sandra Hervás-Stubbs, Asis Palazón, Arantza Azpilikueta, Elixabet Bolaños, Alberto Anel, Julián Pardo, Ignacio Melero

**Affiliations:** 1 CIMA, Gene therapy and Hepatology Unit, University of Navarra, Pamplona, Navarra, Spain; 2 Departamento de Bioquímica y Biología Molecular y Celular, Universidad de Zaragoza, Zaragoza, Spain; 3 Instituto de Investigaciones Sanitarias de Aragón. Departamento de Bioquímica y Biología Molecular y Celular, Fac. Ciencias, Instituto de Nanociencia de Aragón (INA). Fundación Aragón I+D (ARAID) Universidad de Zaragoza, Zaragoza, Spain

## Abstract

**Background:**

Treatment with agonist anti-CD137 (4-1BB) immunostimulatory monoclonal antibodies elicits complete tumor regressions in a number of transplanted hematological and solid malignancies in mice. Rejection is mainly dependent on cytotoxic T lymphocytes (CTL) and IFNγ, although a role for NK cells and dendritic cells has been observed in some tumor models. Rejection of EG7-derived thymomas has been shown to be CTL-dependent but not NK-dependent.

**Findings:**

In this therapeutic setting, we show that both the perforin-granzyme and FasL effector systems are readily expressed by CD8^+^ T lymphocytes infiltrating the EG7 lymphomas which are undergoing rejection. Using knock-out mice, we demonstrate that both effector cytolytic systems are involved in the execution of complete immune rejections against EG7 established tumors. In accordance, EG7 tumor cells were susceptible *in vitro* to both killing mechanisms acting in a synergistic fashion.

**Conclusions:**

CD137-elicited rejection of EG7-derived tumors involves the interplay of at least two final effector cytolytic mechanisms that act in cooperation.

## Findings

### Introduction

CD137 agonists hold promise to augment antitumor immune responses in a clinically significant fashion [1] and two fully human monoclonal antibodies (mAbs) are currently undergoing clinical development (BMS-663513 and PFZ-05082566). Hematological malignancies are not exception to the therapeutic effects of anti-CD137 mAbs and activity has been reported on experimental models of lymphoma, myeloma and mastocytomas [2-4]. The mechanism of action depends mainly on cytolytic T lymphocytes (CTLs) since depletion of CD8β T cells completely abrogates the therapeutic effect [5]. The train of events is complex and needs antigen priming by dendritic cells [5] and in some tumor models the participation of natural killer (NK) lymphocytes as observed in selective depletion experiments [6]. More recently, evidence has been published in the sense that anti-CD137 mAb enhances NK-mediated antibody-dependent cell-mediated cytotoxicity (ADCC) [7,8], in a way that can be exploited to enhance the antitumor activity of Herceptin and Rituximab.

Evidence has been reported showing that activated CD8^+^ tumor infiltrating lymphocytes (TILs) express CD137 [9] and therefore are amenable to receive artificial costimulation by agonist anti-CD137 mAbs within the malignant tissue microenvironment. The execution of tumor rejection requires production of interferon (IFN) γ by CTLs as demonstrated by neutralizing mAbs [10] and with T cells derived from IFNγ^-/-^ mice [10]. However, little is known about the final effector mechanisms that mediate tumor cell killing. CTLs and NK cells may kill using perforin-granzyme, FasL and TNF-related apoptosis inducing ligand (TRAIL) as the executioner molecules [11-14]. Experiments performed in the EG7 tumor model whose successful treatment does not require NK cells [5] clearly show that both the cytolytic granule and the FasL-mediated killing mechanisms were synergistically involved in achieving complete rejections of these lymphomas.

## Results and discussion

### Perforin, granzymes A and B and FasL are involved in tumor rejection elicited by anti-CD137 mAbs

**Figure 1:**
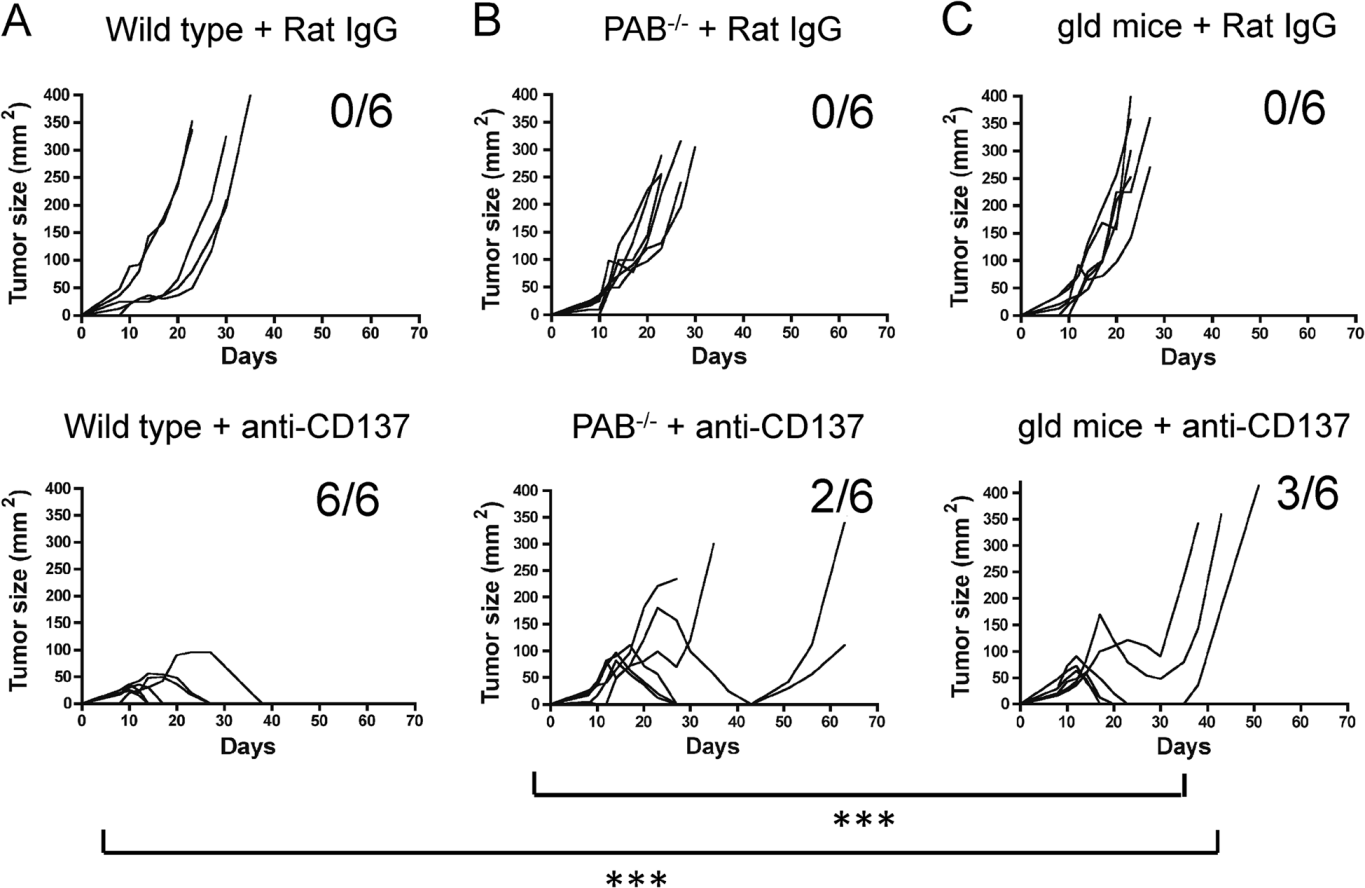
**Both perforin-granzyme and FasL pathways contribute to rejection of EG7 tumors upon treatment with anti-CD137 mAbs.** Wild type (**A**), perforin and granzyme A and B knockout (PAB^-/-^) (**B**) and FasL-mutant gld (**C**) mice were injected s.c. with 5 × 10^5^ EG7 tumor cells and treated i.p. with 100 μg of control Rat IgG or anti-CD137 mAb on days 8, 10, 12 and 14 after tumor cell challenge. Mean tumor diameters were sequentially measured 2-3 times per week. 6 mice per group were included. Statistical comparisons were performed using a nonlinear regression statistical method (Y= (MaxVol * exp(X-TimeO))/( 1 + exp((X-TimeO)/RateGrowth)) with GraphPad software. ***, P<0.001 were considered statistically significant.

As previously published, tumors derived from the EG7 cell line (EL4 stably transfected with ovalbumin [15]) are readily rejected following treatment with anti-CD137 mAb [5]. Treatment of 8-day established tumors with 1D8 mAb achieved complete rejections in six out of six tumors, while the tumors in the control group lethally progressed upon treatment with irrelevant rat IgG (Figure 1A).

Experiments performed in perforin and granzyme A and B triple knockout mice (PAB^-/-^) indicated that although the therapeutic activity was reduced, a residual beneficial effect remained, resulting in two out of six complete rejections (Figure 1B). Conceivably, the FasL-Fas route could also be involved in the execution of rejection by CTLs. Indeed, performing the experiment in mice deficient for FasL (gld mice) also resulted in partial loss of the immunotherapeutic activity of anti-CD137 mAb (Figure 1C). These results are interpreted in the sense that pore-forming and granzyme entrance to malignant cells need to be complemented by FasL-mediated induction of apoptosis in order to optimally achieve tumor rejection.

**Figure 2:**
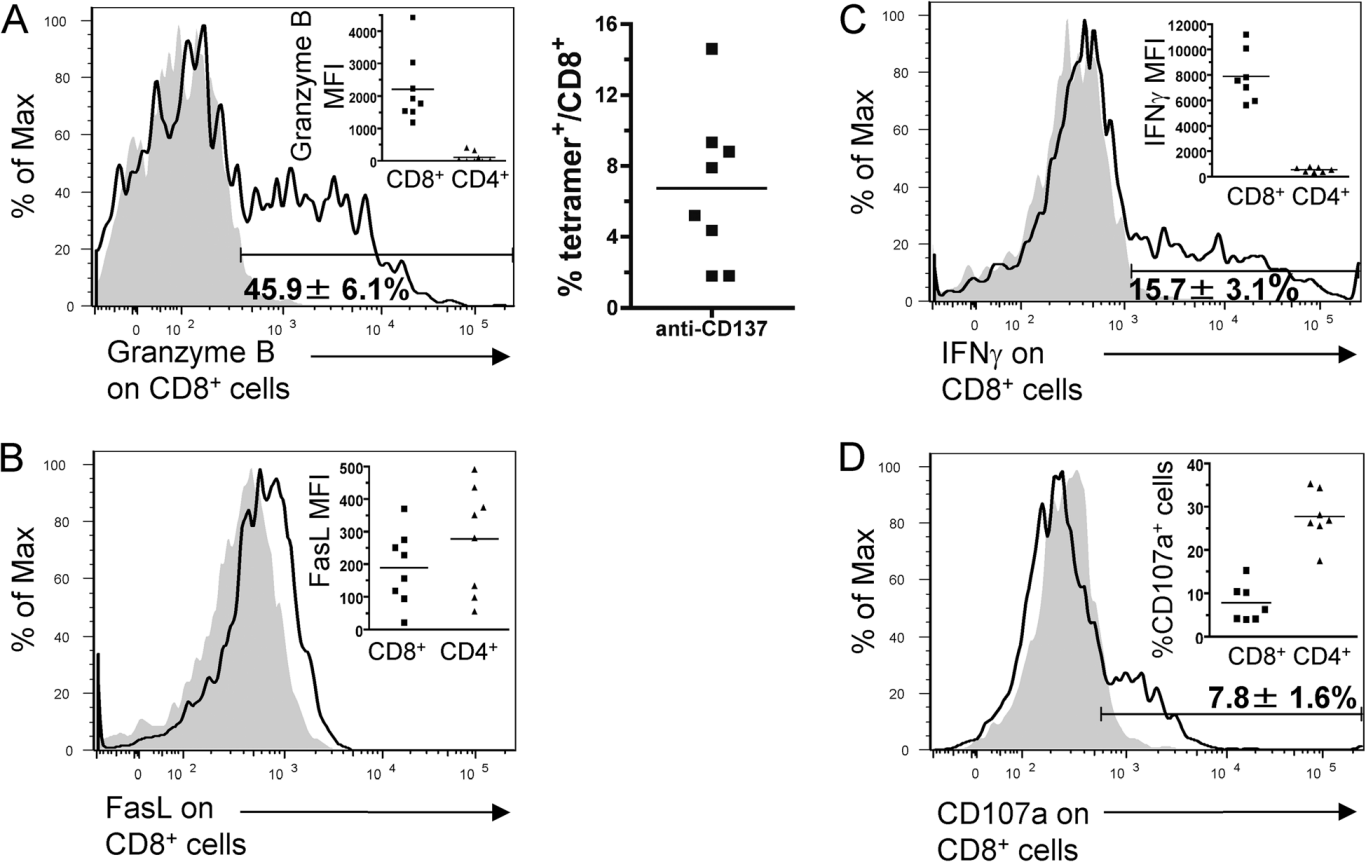
**The cytotoxic mechanisms responsible for EG7 tumor rejection are present in TILs after treatment with anti-CD137 mAb.** Mice were challenged s.c. with 5 × 10^5^ EG7 cells and treated i.p. with anti-CD137 mAb on days 9 and 11. Two days later, tumors were removed and TILs were analyzed by flow cytometry for intracellular granzyme B (**A** left) and percentage of H-2K^b^-SIINFEKL tetramer^+^ cells (**A** right), surface FasL (**B**), intracellular IFNγ (**C**) and surface CD107a (**D**) expression on gated CD8^+^ and CD4^+^ T lymphocytes. Histograms show CD8^+^ gated T cells that come from a representative experiment. Grey histograms represent isotype-matched control antibody stainings and open histograms protein-specific surface or intracellular stainings from representative cases. The insets in each histogram show the mean fluorescense intensity (MFI) after subtracting the background staining or the percentage of CD107a positive cells (**D**) for the indicated immunostainings on CD8^+^ and CD4^+^ T cells from individual tumors. The experiment was performed with 7-8 mice per group and mean±SEM is included in each histogram.

Next, we explored if both killing mechanisms were available in T cells at the site of tumor rejection. As can be seen in Figure 2A, expression of intracellular granzyme B is observed in a good number of CD8^+^ TILs but not in the CD4^+^ counterparts (Figure 2A left). It is of note that 6.7 ± 1.5% of CD8^+^ TILs stained positive with H-2K^b^-SIINFEKL tetramers clearly indicating the presence of antigen specificity in the lymphocyte infiltrate (Figure 2A right). Perforin expression in cytolytic granules could not be explored due to the lack of satisfactory mAbs for staining this protein in mice. FasL (CD95L) was detected on the surface of both CD8^+^ and CD4^+^ TILs (Figure 2B).

Previously published evidence had shown that CTL production of IFNγ was required for tumor rejection [10] and we observed that 15.7 ± 3.1% of CD8^+^ TILs intracellularly express this cytokine without need for any *ex vivo* restimulation of the intratumoral lymphocytes (Figure 2C).

CD107a (LAMP.1) is a cytolytic granule transmembrane protein which emerges at killing synapses and remains transiently on the plasma membrane of effector cells. 7.8 ± 1.6% of CD8^+^ TILs were caught expressing surface CD107a, thus demonstrating that they were actively degranulating at the time of tumor harvest (Figure 2D). The fact that they are holding this “smoking gun” tells of their active participation in cytotoxicity and tumor rejection. In a separate experiment we were able to gate onto T lymphocytes stained with CD8^+^ and H^–2^ K^b^-SIINFEKL tetramers and analyze the expression of effector molecules in this subset. As can be seen in Additional file 1: Figure S1, these CTLs co-express Granzyme B, IFNγ and CD107a.

### EG7 tumor cells are synergistically killed by the perforin-granzyme and the FasL pathways

**Figure 3:**
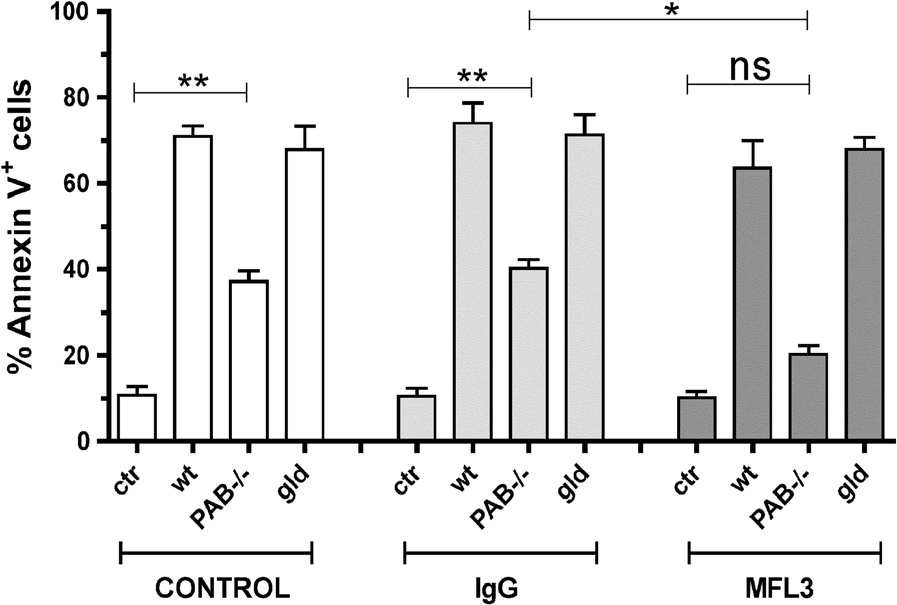
**Fas-FasL pathway and perforin-granzyme machineries synergistically kill EG7 cell line.** 5 × 10^4^ EG7 cells were pre-pulsed with LCMV gp33 peptide and cocultured in vitro for 4 h with 5 × 10^5^ CD8^+^ T cells from LCMV immunized wild type (WT), perforin and granzymes A and B knockout (PAB^-/-^) or gld mice in the presence or absence of 1 μg/ml of FasL blocking antibody (MFL3). Cell death was determined by annexin V staining in CD8 negative population by flow cytometry. Ctr, control with target cells without gp33 peptide. Data in the graphs are represented as mean±SEM of three independent experiments. Statistical comparisons were performed using Student’s t test with GraphPad software. *, *P*<0.05; **, *P*<0.01; ***, P<0.001; ns, no significant. P<0.05 were considered significant.

EG7 cells express surface Fas (Additional file 2: Figure S2A inset) and hence anti-Fas agonistic antibody kills these cells in 18 h as happens in Fas-transfected L1210 cells but not in control untransfected cells. To assess the involvement of these pathways in CTL killing of EG7 cells, anti-lymphocytic choriomeningitis virus (LCMV) derived gp33 CTLs that are directed to a peptide presented by H-2K^b^ were elicited by immunization with LCMV-WE virus. Recovered CD8^+^ splenocytes from immunized wild type (WT) mice 8 days after infections showed 71% killing at 10:1 effector:target ratio on EG7 cells pulsed with the gp33 synthetic peptide (Figure 3A). Under these experimental conditions optimal immunization allows us to focus on the effector phase of CTL activity. This activity was only reduced by about one half when immunized mice were deficient in PAB. Conversely, FasL neutralization with MFL3 mAb during the 4 h cytotoxicity experiment did not hamper killing. Interestingly, this anti-FasL mAb completely abolished cytotoxicity when used on effector CTL from PAB^-/-^ mice. CTLs from gld mice were as effective as WT mice in accordance with the results with FasL single blockade and, as expected, the MFL3 mAb did not have any effect under these conditions, further confirming the selectivity of the blocking mAb.

Therefore at least under these conditions, FasL and the cytolytic granule machinery seem to synergistically operate to bring about death of these lymphoma cells. TRAIL engagement of its death promoting receptors could have also been involved, but this was ruled out since EG7 is not killed by 1μg of a recombinant form of TRAIL that readily killed Jurkat cells as a positive control (Additional file 2: Figure S2B).

## Materials and methods

### Mice and cell lines

C57BL/6 wild type mice (6-8 weeks old) were purchased from Harlan Laboratories. Mice deficient for perforin and granzymes A and B (PAB^-/-^[16]) or with a mutation in FasL (gld mice) were a kind gift of Markus Simon (Max-Planck Institute of Immunobiology, Freiburg, Germany). PAB^-/-^ and gld mice were bred into the BL/6 background. Animal experimentation followed FELASA guidelines and approval of the Ethics Committee for Animal Experimentation from CITA (2011-01) and University of Navarra (study number 066/10). The murine thymoma cell line EG7 was a kind gift from Dr. Claude Leclerc (Institut Pasteur, Paris, France) and was authenticated using the master cell banks by RADIL (Case number: 6592-2012). Jurkat cells, L1210 and Fas-transfected L1210 have been described [17,18].

### *In vivo* tumor growth

5 × 10^5^ EG7 cells were subcutaneously injected into the flank of WT, PAB^-/-^ or gld mice. Mice were intraperitoneally treated with 100 μg/dose of anti-CD137 or control Rat IgG on days 8, 10, 12 and 14 following tumor cell inoculation. Rat IgG antibody was purchased from Sigma-Aldrich and anti-CD137 clon 1D8 [2] was from Bristol-Myers Squibb (Lawrenceville, NJ). Mice and tumor size were monitored twice a week and mice were sacrificed when the tumor size reached 300 mm^2^.

### Preparation of cell suspensions from EG7 tumors

Tumors were excised and incubated for 15 minutes at 37°C in a solution containing Collagenase-D+DNase-I (Roche) in RPMI. Afterwards, tumors were disrupted mechanically and passed through a 70-μm cell strainer (BD Falcon). Erythrocytes from cell suspensions were lysed with potassium ammonium chloride lysing buffer.

### Flow cytometry

Single-cell suspensions were pretreated with FcR-Block (anti-CD16/32 clone 2.4G2). Anti-CD3ϵ, anti-CD8α, anti-CD4, anti-FasL, anti-IFNγ, anti-granzymeB and isotype Rat IgG and Hamster IgG isotype controls were all purchased from Biolegend; anti-CD107a, anti-Fas, mouse IgG isotype control and Apoptosis Detection Kit-I were from BD Pharmingen; and anti-TRAIL mAb was from eBioscience. H-2K^b^-SIINFEKL tetramers were purchased from Beckman Coulter. For intracellular stainings, cells were fixed and permeabilized with Cytofix/Cytoperm (BD Biosciences). Cells were studied with FACSCanto II or FACSCalibur and were analyzed using FlowJo (TreeStar software). Specific mean fluorescense intensity (MFI) and percentage values in graphs are represented after subtraction of the control isotype-matched signal.

### Fas and TRAIL induced cell death assays

10^5^ cells were cultured with human recombinant TRAIL, purified anti-Fas antibody (clone Jo2) or isotype control (both from BD Biosciences) for 18 h. Human recombinant TRAIL functional on human and mouse receptors was produced in *E.coli* and purified as previously described [19]. Cell death was analyzed by Annexin V and 7-aminoactinomycin D (7-AAD) double staining.

### Generation of *ex vivo* gp33-specific CD8^+^ cells

Mice were infected with 10^5^ pfu LCMV-WE i.p. as described [17]. On day eight after infection, CD8^+^ cells were positively selected from spleen using anti-CD8-MicroBeads (Miltenyi Biotec). Purity of selected CD8^+^ cells was higher than 95%.

### *Ex vivo* cytotoxicity assay

Target cells were pre-incubated with the LCMV-immunodominant peptide gp33 (acquired from Neosystem Laboratory) and effector CD8^+^ cells were stained with CellTracker Green (Invitrogen). Effector and target cells were incubated at a ratio of 10:1 (effector:target) during 4 hours. In certain experiment anti-FasL blocking antibody (clone MFL3) (BD Biosciences) was added during the 4 h cytotoxicity assay. Gated CellTracker Green-negative targets were analyzed for annexin V and 7-AAD double staining.

## Conclusions

As a whole, we have demonstrated using a tumor model amenable to successful immunotherapy with anti-CD137 mAb that the FasL and perforin-granzyme killing machineries act non-redundantly and synergistically to execute complete tumor rejections upon therapy with agonist anti-CD137 mAb.

## Abbreviations

mAb: Monoclonal antibody; CTL: Cytotoxic T lymphocyte; NK: Natural killer; IFN: Interferon; ADCC: Antibody-dependent cell-mediated cytotoxixity; TIL: Tumor infiltrating lymphocyte; TRAIL: TNF-related apoptosis inducing ligand; PAB: Perforin and granzyme A and B; LCMV: Lymphocoriomeningitis virus; WT: Wild type; MFI: Mean fluorescense intensity; 7-AAD: 7- Aminoactinomycin D.

## Competing interests

IM has received research grants and consultant honoraria from Bristol Myers Squibb. The rest of the authors do not have conflict of interests to declare.

## Authors’ contributions

IM and JP were the main investigators and take primary responsibility for the paper. AMK and AP performed the in vivo experiments, AMK, EC, AA and EB carried out the in vitro experiments. The tumor infiltration analysis was performed by AMK and SHS. SHS, AA, JP and IM co-ordinated the research and performed laboratory work. IM and AMK wrote the paper. All authors read and approved the final manuscript.

## Supplementary Material

Additional file 1: Figure S1 Experiments as in figure 2 but in this case TILs from five EG7 tumors were pooled and stained with the H-^2^K^b^ SIINFEKL tetramer. Gated CD8^+^ Tetramer+ T lymphocytes were analyzed for expression of the indicated effector molecules as depicted in the corresponding histograms. Click here for file

Additional file 2: Figure S2 The EG7 cell line is susceptible to being killed by anti-Fas antibody but not by recombinant TRAIL. (A) 1 × 10^5^ EG7 cells per well were cultured 18 h with 5 μg/ml of soluble anti-Fas antibody. Cell death was determined by annexin V staining by flow cytometry. The inset histogram shows surface Fas expression by the EG7 cell line. The gray histogram represents isotype-matched control antibody and open histogram Fas-specific surface staining. (B) EG7 or positive control Jurkat cells were incubated for 18 hours with 0 to 1 μg/ml human recombinant TRAIL (hrTRAIL) and cell death was analyzed by flow cytometry using 7-AAD and annexin V staining. Dead cells are represented as the percentage of both Annexin V single positive and Annexin V/7-AAD double positive. Data in the graphs are represented as mean±SEM of three independent experiments. Statistical comparisons were performed using Student’s t test with GraphPad software. *, *P*<0.05; **, *P*<0.01; ***, P<0.001; ns, no significant. P<0.05 were considered significant. Click here for file
