# Supplementary figures and images for: Essential complicity of perforin-granzyme and FAS-L mechanisms to achieve tumor rejection following treatment with anti-CD137 mAb
Source: J Immunother Cancer. 2013 May 29;1:3. doi: 10.1186/2051-1426-1-3 (PMC3987045; doi:10.1186/2051-1426-1-3)

## Slide 1
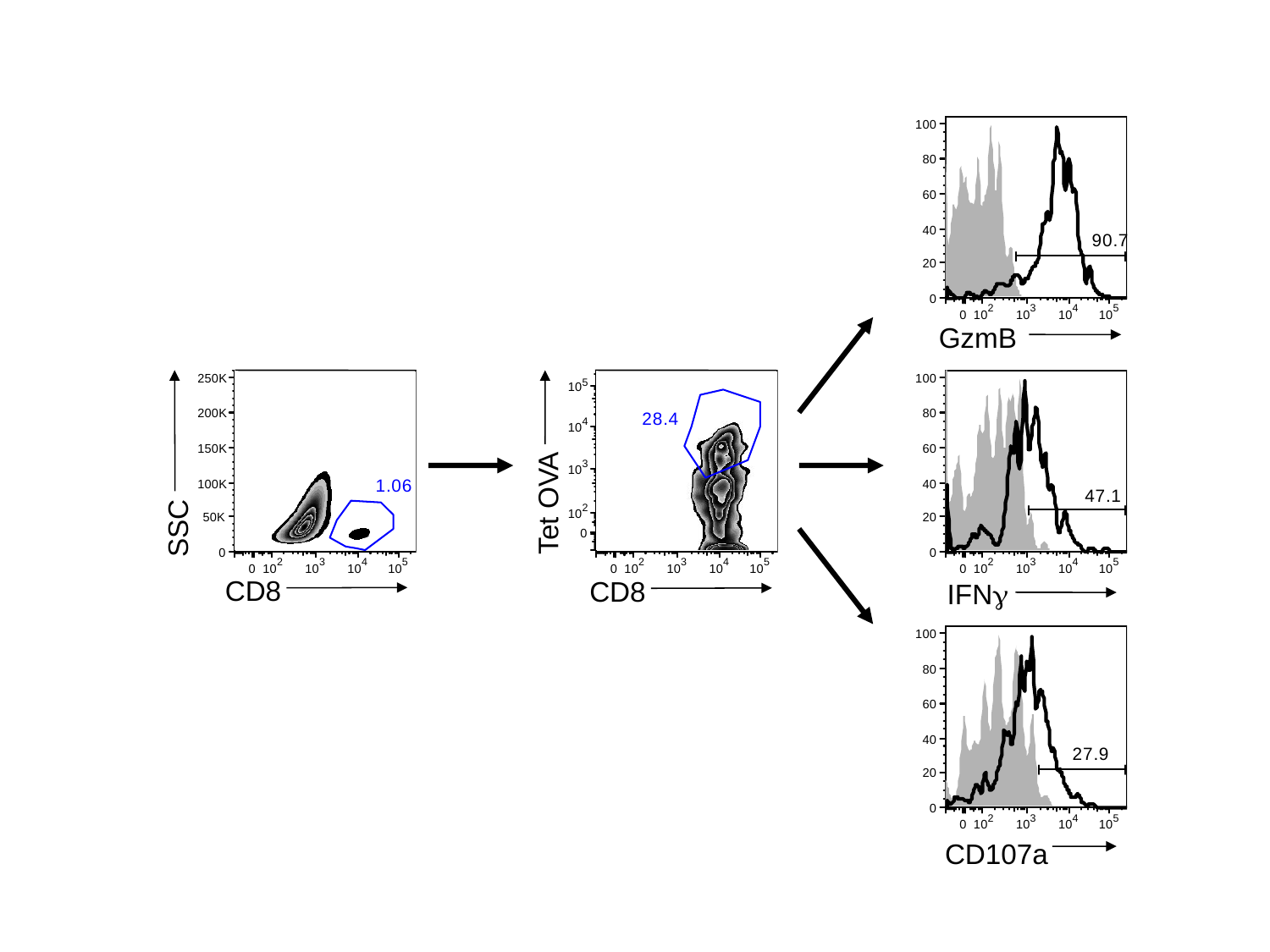

GzmB
Tet OVA
SSC
IFNγ
CD8
CD8
CD107a

Supplement: Additional file 1: Figure S1 — Experiments as in figure 2 but in this case TILs from five EG7 tumors were pooled and stained with the H-2Kb SIINFEKL tetramer. Gated CD8+ Tetramer+ T lymphocytes were analyzed for expression of the indicated effector molecules as depicted in the corresponding histograms. [file 2051-1426-1-3-S1.ppt]

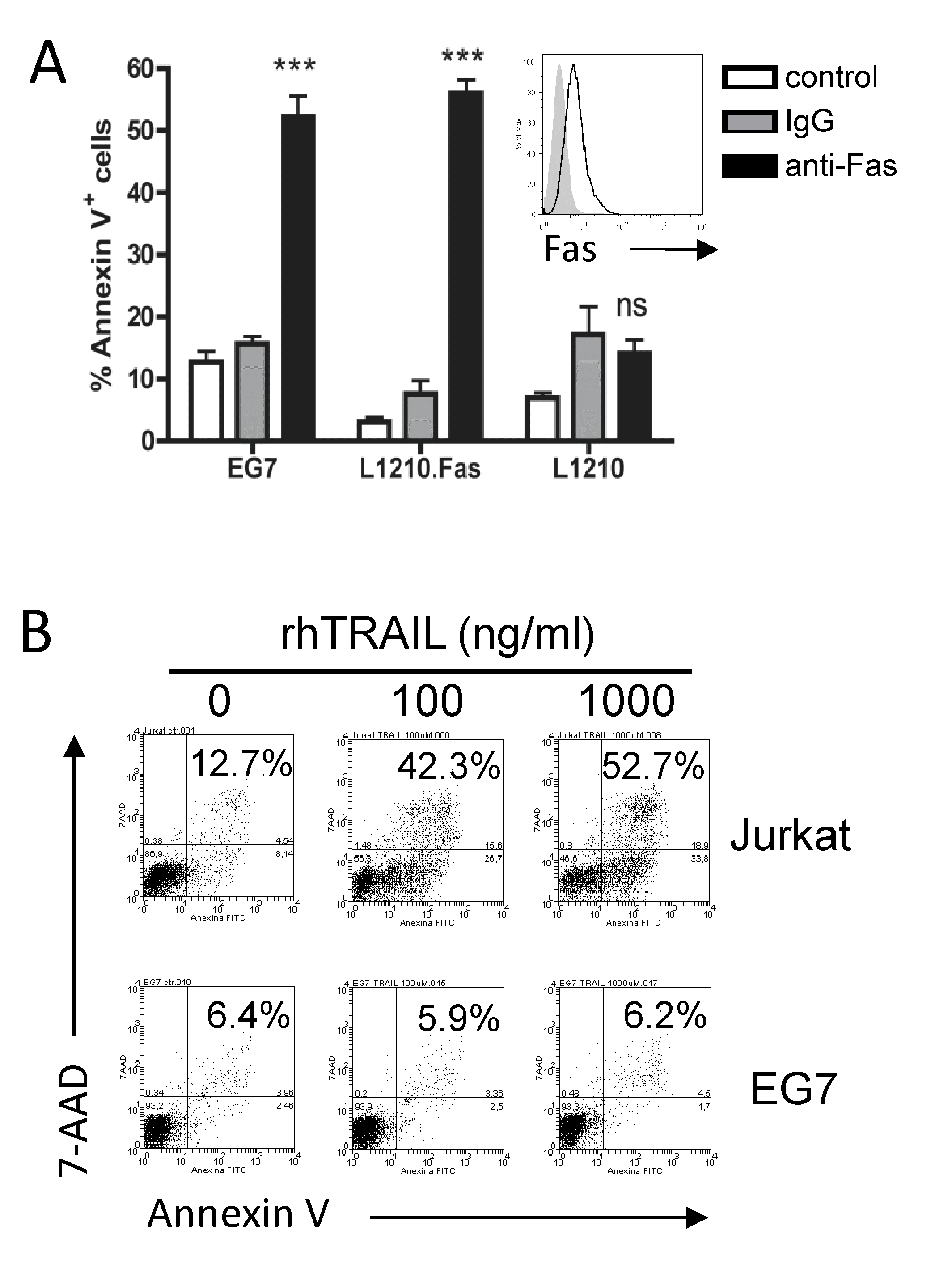

Supplement: Additional file 2: Figure S2 — The EG7 cell line is susceptible to being killed by anti-Fas antibody but not by recombinant TRAIL. (A) 1 × 10^5^ EG7 cells per well were cultured 18 h with 5 μg/ml of soluble anti-Fas antibody. Cell death was determined by annexin V staining by flow cytometry. The inset histogram shows surface Fas expression by the EG7 cell line. The gray histogram represents isotype-matched control antibody and open histogram Fas-specific surface staining. (B) EG7 or positive control Jurkat cells were incubated for 18 hours with 0 to 1 μg/ml human recombinant TRAIL (hrTRAIL) and cell death was analyzed by flow cytometry using 7-AAD and annexin V staining. Dead cells are represented as the percentage of both Annexin V single positive and Annexin V/7-AAD double positive. Data in the graphs are represented as mean±SEM of three independent experiments. Statistical comparisons were performed using Student’s t test with GraphPad software. *, P<0.05; **, P<0.01; ***, P<0.001; ns, no significant. P<0.05 were considered significant. [file 2051-1426-1-3-S2.png]
